# Oral–gut strain sharing after Roux-en-Y gastric bypass: A canonical oral microbiome signature in the gut linked to hepatic and glycemic remodeling

**DOI:** 10.1080/29933935.2026.2693432

**Published:** 2026-07-22

**Authors:** Bas Voermans, Koen Prange, Sjoerd Bruin, Yair Acherman, Egija Zaura, Max Nieuwdorp, Victor Gerdes

**Affiliations:** a Department of Internal and Vascular Medicine, Amsterdam Universitair Medische Centra, Amsterdam, The Netherlands; b Department of Bariatric Surgery, Spaarne Gasthuis Hospital, Hoofddorp, The Netherlands; c Department of Preventive Dentistry, Academisch Centrum Tandheelkunde Amsterdam, University of Amsterdam and Vrije Universiteit Amsterdam, Amsterdam, The Netherlands; d Department of Gastroenterology, Amsterdam Universitair Medische Centra, Amsterdam, The Netherlands; e Department of Internal Medicine, Spaarne Gasthuis, Hoofddorp, The Netherlands

**Keywords:** Bariatric surgery, oral microbiota, gut microbiota, oral-fecal microbiota transfer, metabolic disease

## Abstract

Roux-en-Y gastric bypass (RYGB) induces durable weight loss and metabolic improvement, but the role of oral microbiota in shaping postsurgical gut ecology and metabolic outcomes is unclear. We examined whether RYGB promotes transfer and expansion of oral strains in the distal gut and how these relate to hepatic and glycemic health. In 25 patients from a longitudinal RYGB cohort, paired oral and fecal samples were collected before and 12 months after surgery. We examined the presence, abundance, and structure of cohort-specific canonical oral strains in the gut, and assessed α/β-diversity, cross-site correlations, and clinical associations using univariate tests and linear models. Machine-learning models evaluated the prognostic value of oral canonical strains for hepatic and glycemic outcomes. Oral taxon richness increased after RYGB, while Shannon diversity and individual signatures remained stable. In the gut, canonical oral strains expanded: shared oral-gut strains and their summed abundance rose significantly, converging into a reproducible post-RYGB niche. Abundance and fold change of oral-canonical strains associated with FIB-4, ASAT and fasting glucose, and predictive models suggested a prognostic signal for fasting glucose, TBF% and HbA1c. RYGB is associated with reproducible, strain-level enrichment of oral microbiota in the gut, with links to hepatic and glycemic outcomes.

## Graphical abstract

**Figure:**
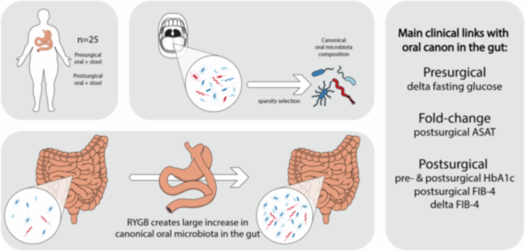

## Introduction

Obesity is a global epidemic associated with significant health risks, including type 2 diabetes (T2D), cardiovascular diseases, and metabolic disorders. Despite the promise of lifestyle interventions and anti-obesity medications, bariatric surgery remains the gold standard for achieving significant and sustained weight loss.^1^ Among the surgical options, Roux-en-Y gastric bypass (RYGB) has demonstrated long-term efficacy in treating obesity and its comorbidities, such as metabolic dysfunction-associated steatotic liver disease (MASLD) and T2D.^2^ ^,^ ^3^ Beyond its mechanistic and hormonal effects, RYGB induces profound changes in the gut microbiome, which are increasingly recognized as critical mediators of its metabolic benefits.^4^

The gut microbiome, a complex ecosystem of microorganisms inhabiting the gastrointestinal tract, plays a substantial role in human health.^5^ It influences key metabolic processes, including short-chain fatty acid (SCFA) metabolism,^6^ bile acid transformation,^7^ and gut hormone regulation.^8^ RYGB has been shown to alter the composition and function of the gut microbiome, with notable shifts in microbial diversity and the relative abundance of specific taxa. For instance, increases in genera such as *Veillonella*, *Streptococcus*, and *Akkermansia muciniphila* have been consistently observed postsurgery, alongside changes in microbial phyla like Bacillota, Bacteroidota, Actinomycetota, and Pseudomonadota.^9^ ^,^ ^10^ These microbial alterations are associated with improved cardiometabolic outcomes, including enhanced gut hormone profiles and bile acid metabolism.^4^

A recurring theme in post-RYGB microbiome studies is the upregulation of facultative anaerobic microbes, many of which are traditionally found in the oral cavity.^4^ These microbes, which can thrive in both oxygen-rich and oxygen-depleted environments, appear to gain a competitive advantage in the altered gut environment following surgery.^11^ ^,^ ^12^ This shift may have implications for weight loss and metabolic improvements, as facultative anaerobes are thought to influence host energy balance and inflammation.^13^ ^,^ ^14^

Many studies have commented on the increased abundance of oral microbes in the gut microbiome following RYGB.^9^ ^,^ ^15-18^ However, these studies have largely been limited to observations of compositional changes and have not demonstrated the direct transfer of oral microbes to the gut. For example, many facultative anaerobic microbes are shared across the proximal, oxygenated parts of the gastrointestinal tract.^19^ This study addresses this gap by employing matched strain-level analysis of both the oral and fecal microbiota to provide stronger evidence of microbial transfer from the oral cavity to the gut. This novel approach allows for a more precise understanding of the dynamics of microbial populations and their potential role in the metabolic benefits observed after RYGB.

The current study builds on this foundation by exploring the role of facultative anaerobic microbes, particularly those originating from the oral cavity, in the postsurgical gut microbiome. By examining strain-level changes and their associations with clinical outcomes, this work aims to shed light on the mechanisms underlying the metabolic benefits of RYGB.

## Methods

### Study design and participants

This study is part of the BARIA cohort.^20^ The BARIA cohort is a prospective observational cohort study that includes patients with obesity who are scheduled for RYGB at Spaarne Gasthuis in Hoofddorp (the Netherlands). The study was approved by the Medical Ethics Review Committee of the Amsterdam UMC (approval code: NL55755.018.15, date: 27-05-2020). All participants provided written informed consent. From the pool of patients who had both pre- and postsurgical oral and fecal samples available, 25 were randomly selected for this study. Fecal and oral microbiome samples were collected from patients undergoing surgery before and 12 months after RYGB. Fecal samples were collected in sterile containers and stored at −80 °C until further processing. Oral samples were collected using a sterile swab, which was wiped over the tongue base and was subsequently placed in a sterile tube and stored at −80 °C until further processing.

The comorbidities T2D, MASLD, dyslipidemia, and hypertension were assessed using different approaches depending on the timepoint and the specific condition. At baseline, the presence of T2D, dyslipidemia, and hypertension was determined by patient self-report and confirmed based on clinical information using established guidelines.^21-23^ At 12-month follow-up, dyslipidemia and hypertension were again assessed by self-report. Postsurgical T2D was not evaluated by self-report; instead, the World Health Organization diagnostic criterion for fasting blood glucose was applied.^22^ MASLD was assessed using liver biopsies taken during the RYGB procedure, and diagnoses were established using tandem scoring. For all comorbidities except MASLD, remission was defined as the condition being present at baseline but absent at the 12-month timepoint. Metabolic Dysfunction–Associated Fibrosis 5 (MAF-5) and Fibrosis 4 (FIB-4) scores were used as noninvasive tests (NITs) for MASLD.^24^ MAF-5 was chosen in addition to FIB-4 since it is an age-independent NIT validated in a Dutch population.^25^

### Shotgun metagenomics sequencing and profiling

Total genomic DNA was extracted using a modified version of the IHMS DNA extraction protocol Q.^26^ In brief, samples were extracted in Lysing Matrix E tubes (MP Biomedicals) containing ASL buffer (Qiagen). Lysis was obtained after homogenization by vortexing for 2 minutes, followed by two cycles of heating at 90 °C for 10 minutes and three bursts of bead beating at 5.5 m/s for 60 seconds in a FastPrep-24 instrument (MP Biomedicals). After each bead-beating burst, samples were placed on ice for 5 minutes. The supernatants containing DNA were collected after the two cycles by centrifugation at 4 °C. Supernatants from the two centrifugation steps were pooled, and a 600 μL aliquot from each sample was purified using the QIAamp DNA Mini kit (QIAGEN) in the QIAcube instrument (QIAGEN) using the procedure for human DNA analysis. Samples were eluted in 200 μL of AE buffer (10 mM Tris-Cl, 0.5 mM EDTA, pH 9.0). Libraries for shotgun metagenomic sequencing were prepared by a PCR-free method; library preparation and sequencing were performed at Novogene (Cambridge, UK) on a HiSeq instrument (Illumina) with 150-bp paired-end reads and 6 Gb data per sample.

Quality and adapter trimming of raw reads was performed with fastp,^27^ and human reads were filtered out by mapping reads to reference human genome hg38 using Bowtie 2.^28^ ^,^ ^29^ Metagenomic sequencing data was profiled using MetaPhlan4 to obtain taxonomic profiles.^30^ Strain-level profiling was performed using StrainPhlan3.^31^

### Statistical analyzes

All analyzes were performed in Python 3.13.11. Shannon index was calculated using the following formula: H = $-\sum_{i=1}^{R}p_{i}\;\ln{(p}_{i})$ , where $p_{i}$ is the proportion of the i -th species in the sample. Richness was defined as the total number of identified strains present in the sample. Beta diversities between samples and subsequent PCoA ordinations were performed with the scikit-bio 0.7.1 package using Bray‒Curtis dissimilarity for the distance metric. Statistical differences between groups were calculated using the Mann‒Whitney U (MWU) test for unpaired samples and the Wilcoxon signed-rank test for paired samples. Correlations were calculated using Spearman's rank correlation. Proportion differences of dichotomous outcomes between groups were calculated using a Chi-squared test. Changes in comorbidities were assessed using a McNemar test. *p*-values were adjusted for multiple testing using the Benjamini‒Hochberg (BH) procedure. Linear models and corresponding *p*-values were created using the ordinary least squares module of the statsmodels 0.14.6 package. Performance metrics were calculated using the scikit-learn 1.8.0 package. Estimation of 95% confidence intervals of the performance metrics were calculated using 100 folds of resampling with replacement.

Plots were created using the matplotlib 3.10.8 and seaborn 0.13.2 packages. Annotations of significance were added using the statannotations 0.7.1 package.

### Selection of the oral canonical strains

For the establishment of what comprises the core oral microbiome in this RYGB cohort, we performed abundance and prevalence selection. This is a recommended step in filtering the oral metagenomic data as false discoveries of species are often found in metagenomic taxonomic profiling.^32^ ^,^ ^33^ To this end, strains (SGBs) detected in at least 20% of oral samples (pre- or postsurgery; nonzero relative abundance) were retained. This prevalence threshold was chosen to exclude sporadic, low-prevalence detections while preserving the dominant community signal. The same 20% prevalence threshold was used for the analysis of the Personalized Responses to Dietary Composition Trial (PREDICT 1) study, a large, deeply phenotyped cohort which used similar methods for metagenomic sequencing and profiling.^34^ We name this set of strains the canonical oral microbiota.

### Machine learning (ML)

In order to assess the predictive multivariate power of the canonical oral microbiota, we employed ML models using the oral canon in the gut as input and clinical characteristics as output. These models work similarly to linear or logistic regression models while being able to handle nonlinear relationships as well as interactions between the strains in the oral canon. In our first ML experiment, we performed a regression analysis on the continuous outcomes. In addition to regression, we performed a second set of ML experiments on the median-split dichotomized continuous variables. As a third and final step, we performed additional ML runs of the best-performing classification experiments with some confounder clinical variables added to create a clinical-microbiome integrated ML model.

All ML analyzes were performed using 100 stratified splits to split training and test data with an extra-trees model architecture as available in scikit-learn 1.8.0. In each split, 20% of the data was reserved for testing purposes only. Hyperparameters were optimized with three-fold cross-validation. Performance was measured using root mean squared error (RMSE) for regression. For dichotomized variables, area under the curve of the receiver operating characteristic curve (ROC–AUC) was used.

In order to assess the significance of our results, we performed a permutation test comprising of another 100 stratified splits with permuted outcomes. In these experiments, all settings were kept equal to the runs where the true labels were used. An MWU test was then used to assess whether the true labels yielded significantly different performance from randomly permuted labels. Furthermore, permutation testing also measures whether there is any data leakage occurring in the ML method for the classification experiments, as an AUC approaching 0.5 signifies random performance of the ML method. If performance on the permuted dichotomized outcomes is greater than 0.5, data leakage would be occurring.

## Results

The baseline characteristics of patients included in this paper are shown in Table 1. Mean ± SD of age at baseline was 48.12 ± 7.5 y, and BMI was 39.1 ± 2.8. 64% of the included patients were female, and 24% were diagnosed with T2D when admitted to the bariatric procedure. An earlier multi-center study compiling baseline characteristics of 5516 Dutch RYGB patients found mean ± SD age of 43.6 ± 10.5 y and a BMI of 39.1 ± 2.8. In addition, 84% of the people admitted to surgery were female, and 21.4% were diagnosed with T2D^35^ No patients reported the use of antibiotics at any of the study timepoints. At baseline, 5 patients used oral glucose-lowering medication; for all five, this medication was discontinued after surgery and was not restarted during the 12-month follow-up period. Use of antihypertensive and glucose-lowering medication per patient is given in Supplemental Table 1. Considering the small sample size of the current study, based on the larger RYGB cohort, we would consider these patients representative of a Dutch RYGB population. Presurgical, postsurgical, and cross-surgical delta of body weight, blood pressure, metabolic markers, and MASLD (NITs) are shown in Table 2. We observed an average TWL% of 30.43 ± 5.84 and a reduction of TBF% of 16.82 ± 6.2 percentage points delta. Moreover, across the panel of metabolic parameters, a marked improvement is seen, indicating a significant enhancement in cardiometabolic health, as is expected after RYGB.

**Table 1.** Baseline characteristics for bariatric surgery patients at inclusion.

Variable		Unit	Mean ± SD [IQR]/*n* (%)
**Demographics and anthropometric measurements**			
N			25
sex (=Female)			16 (64%)
Age		[y]	48.12 ± 7.5 [42–54]
BMI		[kg/m2]	39.1 ± 2.8 [38–41]
Weight		[kg]	117.8 ± 12.5
Waist circumference		[cm]	122.1 ± 11
Total body water		[%]	40.05 ± 5.98 [37.41–43.04]
Total body fat		[%]	45.12 ± 5.98 [41.67–49.01]
Hypertension			11 (44%)
T2D			6 (24%)
PPI use			7 (28%)
**Liver steatosis and fibrosis grades**			
Liver steatosis grade	0		10 (40%)
	1		11 (44%)
	2		2 (8%)
	3		1 (4%)
	unknown		1 (4%)
Liver fibrosis grade	0		4 (16%)
	1		13 (52%)
	2		1 (4%)
	3		0 (0%)
	4		6 (24%)
	unknown		1 (4%)

**Table 2.** Presurgical, postsurgical and deltas of RYGB patient characteristics.

Variable	Unit	Baseline Mean ± SD [IQR]/*n* (%)	1-y follow-up Mean ± SD [IQR]/*n* (%)	*p*-value	Delta Mean ± SD [IQR]/*n* (%)
**Anthropometric measurements**					
TWL	[%]				30.43 ± 5.84 [28.28 ─ 34.40]
BMI	[kg/m^2^]	**39.1 ± 2.7 [37.6 ─ 40.6]**	**27.2 ± 2.4 [25.7 ─ 28.4]**	****	**−11.9 ± 2.5 [−13.2 ─ −11.2]**
Weight	[kg]	**117.8 ± 12.2 [109.0 ─ 127.6]**	**81.9 ± 10.5 [76.0 ─ 87.6]**	****	**−35.9 ± 7.8 [−41.0 ─ −32.4]**
Waist circumference	[cm]	**122.1 ± 10.3 [114.0 ─ 127.0]**	**96.4 ± 9.2 [89.0 ─ 102.0]**	****	**−25.7 ± 8.2 [−30.0 ─ −19.0]**
Total body fat	[%]	**45.3 ± 5.9 [42.7 ─ 49.0]**	**28.5 ± 5.7 [25.6 ─ 31.8]**	****	**−16.8 ± 6.0 [−21.0 ─ −14.4]**
**Blood pressure and hypertension**					
Systolic blood pressure	[mmHg]	**127.2 ± 12.3 [121.0 ─ 135.0]**	**121.1 ± 17.2 [110.0 ─ 130.0]**	*	**−6.1 ± 15.2 [−12.0 ─ −3.0]**
Diastolic blood pressure	[mmHg]	81.9 ± 8.3 [78.0 ─ 88.0]	80.6 ± 9.4 [74.0 ─ 86.0]	ns	−1.4 ± 10.2 [−5.0 ─ 1.0]
Hypertension		14.0 (56.0%)	19.0 (76.0%)	ns	
Hypertension change	Remission (Yes → No)				0 (0%)
	Remain (Yes → Yes)				6 (24%)
	Gain (No → Yes)				0 (0%)
	Absence (No → No)				19 (76%)
**Glucose metabolism**					
Fasting blood glucose	[mmol/L]	**6.18 ± 1.13 [5.48 ─ 6.45]**	**5.11 ± 0.35 [4.97 ─ 5.22]**	***	**−1.07 ± 1.07 [−1.18 ─ −0.4]**
HbA1c	[mmol/mol]	**6.0 ± 0.97 [5.5 ─ 6.1]**	**5.34 ± 0.26 [5.3 ─ 5.4]**	****	**−0.66 ± 0.87 [−0.7 ─ −0.2]**
T2D		**6 (24%)**	**0 (0%)**	*	
T2D change	Remission (Yes → No)				6 (24%)
	Remain (Yes → Yes)				0 (0%)
	Gain (No → Yes)				0 (0%)
	Absence (No → No)				19 (76%)
**Lipid profile and dyslipidaemia**					
Total cholesterol	[mmol/L]	**4.9 ± 0.98 [4.3 ─ 5.5]**	**4.62 ± 0.68 [4.4 ─ 5.1]**	*	**−0.28 ± 0.73 [−0.8 ─ 0.3]**
HDL cholesterol	[mmol/L]	**1.27 ± 0.42 [1.0 ─ 1.4]**	**1.52 ± 0.38 [1.3 ─ 1.7]**	**	**0.25 ± 0.49 [0.18 ─ 0.45]**
LDL cholesterol	[mmol/L]	**3.29 ± 0.96 [2.6 ─ 4.0]**	**2.68 ± 0.67 [2.1 ─ 3.1]**	**	**−0.62 ± 0.76 [−1.1 ─ −0.1]**
Triglycerides	[mmol/L]	**1.55 ± 0.52 [1.15 ─ 1.74]**	**1.01 ± 0.46 [0.6 ─ 1.3]**	****	**−0.54 ± 0.43 [−0.82 ─ −0.24]**
Dyslipidaemia		**18.0 (72.0%)**	**9.0 (36.0%)**	*	
Dyslipidaemia change	Remission (Yes → No)				10 (40%)
	Remain (Yes → Yes)				8 (32%)
	Gain (No → Yes)				1 (4%)
	Absence (No → No)				6 (24%)
**Hepatic markers and MASLD noninvasive tests (NIT)**					
AST	[U/L]	23.76 ± 5.87 [20.0 ─ 24.0]	24.43 ± 6.07 [21.0 ─ 28.0]	ns	0.67 ± 6.92 [−2.0 ─ 6.0]
ALT	[U/L]	30.95 ± 17.78 [22.0 ─ 33.0]	34.71 ± 14.79 [25.0 ─ 40.0]	ns	3.76 ± 19.44 [−2.0 ─ 14.0]
FIB-4		**0.78 ± 0.24 [0.57 ─ 0.99]**	**0.88 ± 0.33 [0.65 ─ 1.04]**	*	**0.1 ± 0.17 [0.04 ─ 0.18]**
MAF-5		**2.2 ± 1.54 [1.05 ─ 2.77]**	**−0.23 ± 1.16 [−0.97 ─ 0.78]**	****	**−2.42 ± 1.13 [−3.26 ─ −1.25]**
**Inflammatory and renal biomarkers**					
CRP	[mg/L]	**6.45 ± 4.49 [3.2 ─ 8.0]**	**1.14 ± 0.79 [1.0 ─ 1.8]**	***	**−5.32 ± 4.38 [−7.75 ─ −2.2]**
Creatinine	[µmol/L]	**67.9 ± 9.19 [62.0 ─ 76.0]**	**63.48 ± 7.65 [60.0 ─ 67.0]**	*	**−4.43 ± 7.64 [−11.0 ─ 0.0]**
Ferritin	[µg/L]	**111.0 ± 87.1 [31.0 ─ 172.0]**	**72.2 ± 50.4 [23.0 ─ 104.0]**	**	**−38.8 ± 55.2 [−51.0 ─ −1.0]**

*p*-value legend: ****: *p* < 0.0001, ***: 0.0001 < *p* < 0.001, **: 0.001 < *p* < 0.01, *: 0.01 < *p* < 0.05. Rows shown in **Bold** indicate a significant cross-surgical change.

### The oral microbiome before and after RYGB

**Figure 1.**
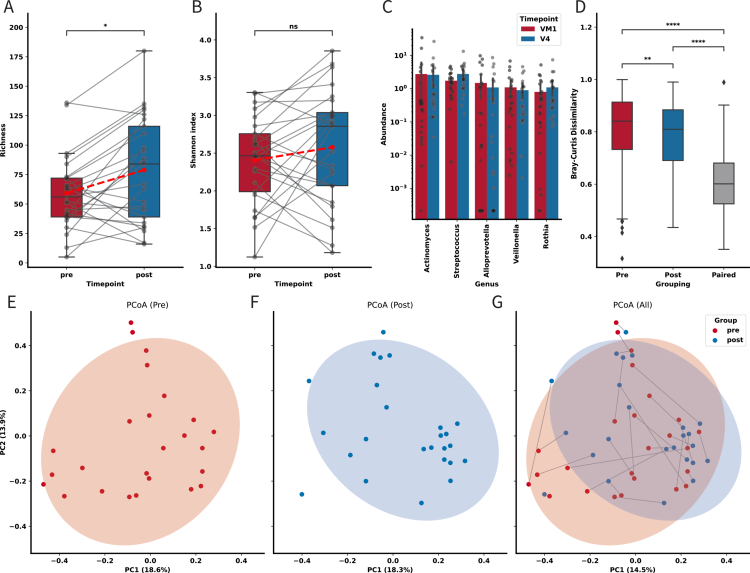
A: Strain-level richness (number of strains) of the oral microbiota before and after RYGB. B: Strain-level Shannon index in the oral microbiota before and after RYGB. C: The top-5 most abundant genera in the oral microbiota before and after RYGB. D: Boxplots of Bray‒Curtis dissimilarities between the oral microbiota of people before surgery, 12 months after surgery, and the intraindividual dissimilarity. E: PCoA plot of the oral microbiota before RYGB. F: PCoA plot of the oral microbiota after RYGB. G: joined PCoA plot of the oral microbiota before and after RYGB, lines connect paired samples.

The oral microbiota was assessed in 25 patients before and 12 months after RYGB. Oral strain richness increased significantly after RYGB from a mean ± SD of 59.28 ± 31.8 strains to 78.88 ± 44.73 strains (MWU *p* = 0.01247, Figure 1A). The Shannon index was unaltered after RYGB, with a mean ± SD of 2.41 ± 0.58 before and 2.58 ± 0.77 after RYGB (MWU *p* = 0.2521, Figure 1B). On a genus level, the most abundant genera in the oral microbiome were *Actinomyces*, *Streptococcus*, *Alloprevotella*, *Veillonella* and *Rothia*. On a strain level, none of the strains were significantly altered after RYGB.

Principal coordinate analysis (PCoA) of Bray‒Curtis dissimilarities (Figure 1E−G) demonstrates a compositional shift in the oral microbiome 12 months after RYGB. Postsurgical samples were more similar to one another than presurgical samples (MWU *p* = 0.0026; Figure 1D). In addition, within-subject (paired pre-post) dissimilarity was significantly lower than the dissimilarity between unmatched pre- and postsurgical samples (MWU *p* < 0.0001; Figure 1D), indicating that surgery induces a community shift while preserving an individual-specific microbial signature.

### The canonical oral microbiome

To enable a robust assessment of oral-gut strain sharing, we first defined a cohort-specific canonical oral microbiome at the strain level. The resulting set comprised 105 of the 423 strains detected (24.8%), yet accounted for 88.5% of the cumulative strain-level relative abundance, indicating that removed low-prevalence strains (present in <20% of samples) contributed minimally to overall community structure.

#### Strain sharing between the oral cavity and the gut

**Figure 2.**
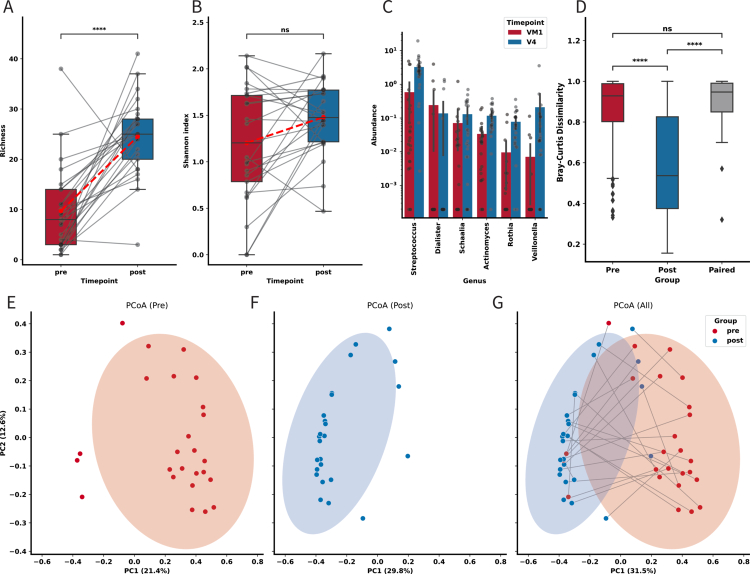
A: Strain-level richness of the oral canon in the gut microbiota before and after RYGB. B: Strain-level Shannon index of the oral canon in the gut microbiota before and after RYGB. C: The top-5 most abundant genera in the oral canon before and after RYGB. D: Comparison of Bray‒Curtis dissimilarities between the oral canon in the gut microbiota of people before surgery, after surgery, and the intraindividual dissimilarity. E: PCoA plot of the oral canon in the gut before RYGB. F: PCoA plot of the oral canon in the gut after RYGB. G: joined PCoA plot of the oral canon in the gut before and after RYGB, lines connect paired samples.

The canonical oral microbiome was used to assess strain sharing between the oral cavity and the gut. Strains that were present in the prevalence-filtered oral dataset were selected in the fecal dataset to represent the canonical oral microbiota in the gut. This resulted in a total of 60 strains that were shared between the oral cavity and the gut. 49 strains of the oral canon were shared before surgery, while 59 strains were shared 12 months after RYGB. The strain richness of the oral canon in the gut increased significantly after RYGB (Wilcoxon *p* = 0.00018, Figure 2A). However, despite the increase in shared strains, the Shannon index within the oral canon in the gut did not change significantly after RYGB (Wilcoxon *p* = 0.052, Figure 2B). The top 5 genera of the shared strains were *Streptococcus*, *Dialister*, *Schaalia*, *Actinomyces* and *Rothia*. After RYGB, the top genera within the oral canon in the gut were *Streptococcus*, *Veillonella*, *Dialister*, *Schaalia* and *Actinomyces*, showing an increase in abundance of *Streptococcus* (BH-adjusted Wilcoxon *p* = 0.0009), *Schaalia* (BH-adjusted Wilcoxon *p* = 0.043), *Actinomyces* (BH-adjusted Wilcoxon *p* = 3.6E-6), *Rothia* (BH-adjusted Wilcoxon *p* = 0.0002) and *Veillonella* (BH-adjusted Wilcoxon *p* = 0.002), and a nonsignificant decrease in relative abundance of *Dialister* (Figure 2C).

Contrary to the microbiota in the oral cavity, the oral canon in the gut showed such a large shift in composition that the pre- and postsurgery samples were clearly distinguishable by eye in a PCoA plot (Figure 2E−G). The strain-level interindividual dissimilarity of the oral canon in the gut after surgery was significantly lower than both the intraindividual dissimilarity and the dissimilarity between samples before surgery (Wilcoxon *p* < 0.0001 and *p* < 0.0001 respectively, Figure 2D). This shows that the oral canon in the gut was more similar between individuals after RYGB than before RYGB, indicating a shift of the oral canon in the gut after RYGB to a specific postsurgical oral canon composition. The intraindividual dissimilarity was not different from the between-samples dissimilarity before RYGB (Wilcoxon *p* = 0.54, Figure 2D).

**Figure 3.**
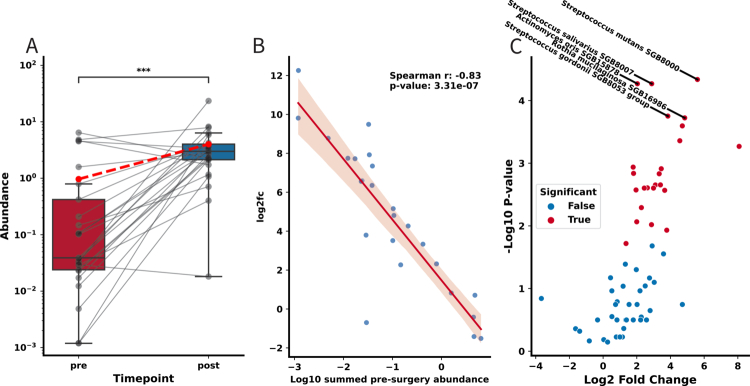
A: Boxplot of the summed abundance of the RYGB oral canon in the gut before and after surgery. B: Scatterplot illustrating the correlation between log2fold-change after surgery and presurgical abundance of the oral canon. Low presurgical abundance of the oral canon is associated with higher fold-change and vice versa. C: Volcano plot of the strain-level log2 fold change of the oral canon in the gut before and after RYGB. The red dots indicate strains that were significantly altered after RYGB (Wilcoxon *p* < 0.05).

The summed abundance of the oral canon in the gut increased with a mean ± SD of log2 fold change of 4.70 ± 3.72 after RYGB (Wilcoxon *p* = 0.0006, Figure 3A). The log2 fold change of the oral canon in the gut was negatively correlated with the presurgical abundance of the oral canon in the gut (Spearman's Rho = −0.83, *p* < 0.0001, Figure 3B). This shows that patients with a low presurgical abundance of the oral canon in the gut had a higher fold change after RYGB than patients with a high presurgical abundance of the oral canon in the gut.

#### Correlations of the oral canon in the oral cavity and the gut

Significant correlation between specific strains of the oral canon in the oral cavity and the gut were found for 8 strains (BH adjusted Spearman's Rho *p* < 0.05). These strains were: *Rothia mucilaginosa* SGB1698, *Schaalia meyeri* SGB1715, *Schaalia odontolytica* SGB1716, *Schaalia sp.* SGB1715, *Actinomyces sp. ph3* SGB17137, *Isoptericola variabilis* SGB1715, *Actinomyces sp. oral taxon 448* SGB1587, *Actinomyces naeslundii* SGB1588. All correlations were positive, indicating that a high abundance of these strains in the oral cavity is associated with a high abundance of the same strain in the gut.

#### Strain-level log2 fold change of the oral canon in the gut

Out of the 60 strains constituting the oral canon and the gut, 24 strains were found to be altered after RYGB (BH-adjusted Wilcoxon *p* < 0.05, Figure 3C). The strains that were significantly altered after RYGB were predominantly from the genera *Streptococcus*, *Actinomyces,* and *Rothia*. All altered strains were increased in relative abundance after surgery. The altered strains represent a mean ± SD of 62% ± 16% of the total abundance of the oral canon in the gut before surgery and 90% ± 16% after surgery, showing that the strains that were significantly altered after RYGB represent a majority of the oral canon in the gut as a whole.

#### Summed abundance of the oral canon in the gut and clinical parameters

**Figure 4.**
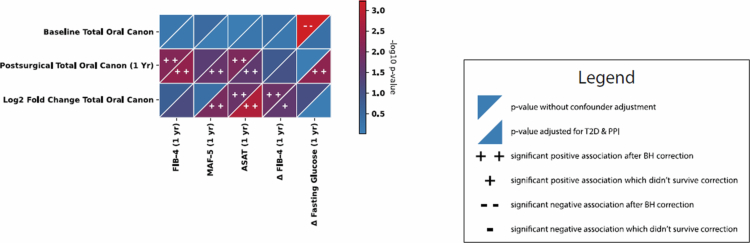
Associations between the summed pre- and postsurgical abundance and fold change of the total oral canon in the gut and clinical parameters.

The summed postsurgical abundance and fold change of the oral canonical strains in the gut as a whole were found to be significantly associated with clinical parameters. In Figure 4, these results are shown both with and without adjustment for baseline T2D and PPI use. These results (Figure 4) show an association between the postsurgical canon and postsurgical markers for hepatic metabolic health FIB-4 and ASAT with and without adjustment for confounders. In addition, the change in abundance of the oral canon was associated with postsurgical ASAT as well. Showing a link between higher postsurgical abundance and change in hepatic metabolic health.

#### The oral canon in the gut and clinical parameters

**Figure 5.**
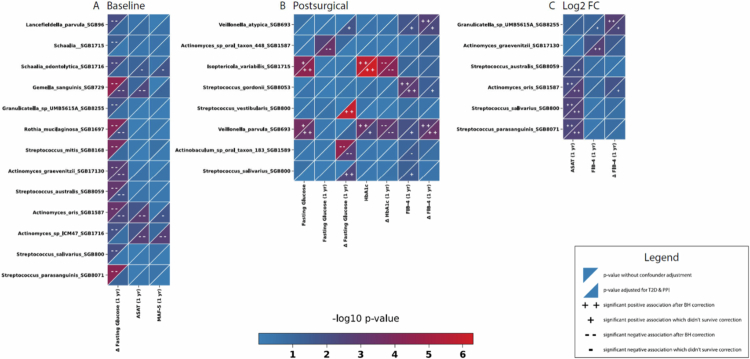
A: Significant associations between the presurgical abundance of the strains in the oral canon in the gut and clinical characteristics after surgery and cross-surgical delta. B: Significant associations between the postsurgical oral canon in the gut and clinical characteristics after surgery and cross-surgical delta. C: Significant associations between the log2fold change of the oral canon in the gut and clinical characteristics after surgery and cross-surgical delta.

Out of the strains in the oral canon, the relative abundance in the gut of 20 specific strains were found to be significantly associated with at least one clinical parameter at one or more timepoint. These parameters consist of presurgical, postsurgical, and change in clinical parameters. The strains found to be associated with the clinical parameters based on their presurgical abundance are shown in Figure 5A. Similarly, those associated with clinical parameters based on their postsurgical abundance and log2fold change are shown in Figure 5B and C, respectively. In a similar fashion as Figure 4, the *p*-values are shown with and without confounder adjustment. As can be observed, several strains are only significantly associated either with or without confounder adjustment. These strains are shown for completeness. However, given the size of this cohort, the focus of this section is positioned on associations that are consistent with and without confounder adjustment. The data presented in Figure 5A−C is presented in table format in supplementary Table 2.

The presurgical abundance of five strains was associated with the change in fasting glucose after RYGB. These microbes form a specific cluster, as the presurgical total abundance of the oral canon as a whole was associated with the delta of fasting glucose. Namely, *Gemella sanguinis SGB729*, *Rothia mucilaginosa* SGB1697, *Actinomyces graevenitzii* SGB17130, *Streptococcus australis* SGB8059, *Actinomyces oris* SGB1587. Thus, higher presurgical abundance of this cluster is associated with better outcome in terms of fasting blood glucose.

The associations of the postsurgical abundance of the strains in the oral canon and clinical characteristics gives a less uniform view, as is shown in Figure 5B. Postoperative *Actinobaculum sp. oral taxon 183* SGB1589 was associated with lower fasting glucose at that same timepoint. *Isoptericola variabilis* SGB1715 after surgery was positively associated with presurgical HbA1c, possibly indicating larger niche for this SGB in patients with higher HbA1c. *Veillonella parvula* SGB693 and *Isoptericola variabilis* SGB1715 were associated with a larger improvement in HbA1c change after RYGB. Finally, two taxa were associated with worse MASLD scores based on FIB-4. Postsurgical *Streptococcus gordonii* SGB8053 was associated with higher postsurgical FIB-4, and higher postsurgical *Veillonella parvula* SGB693 was associated with less improvement in FIB-4.

All strains whose log2fold changes were associated with clinical characteristics indicated associations with poorer hepatic metabolic health. The fold-change of *Streptococcus parasanguinis* SGB8071, *Streptococcus salivarius* SGB800, *Actinomyces oris* SGB1587 were all positively associated with postsurgical ASAT.

### Prediction of postsurgical clinical parameters

In our machine learning experiments, we measured the performance of the gut relative abundance of strains in the oral canon in predicting postsurgical clinical parameters that are of interest for metabolic improvement after surgery. For this reason, the predicted target variables were BMI, TBF%, TWL%, fasting glucose, HbA1c, and MAF-5 at 12 months after surgery. MAF-5 was chosen as an age-independent NIT for MASLD severity as no postsurgical biopsies were available. The features used for the prediction experiments were the presurgical abundance of the oral canon in the gut, the postsurgical abundance of the oral canon in the gut, log2 fold change of the oral canon in the gut, and the presurgical clinical characteristics. For all experiments, individual strains were used, rather than summed abundance.

The regression experiments yielded no statistically significant results, indicating that none of the target variables could be predicted significantly better than random (Table 3). This likely could be attributed to sample size.

**Table 3.** Regression results comparing each target value with a permuted version of itself. None of the results showed a significant difference between true and randomized labels.

	Presurgical				Postsurgical				Log2Fold change			
Target	RMSE	RMSE (permuted)	*p*	*p*_adj	RMSE	RMSE (permuted)	*p*	*p*_adj	RMSE	RMSE (permuted)	*p*	*p*_adj
BMI (1 y)	1.040 ± 0.292	1.031 ± 0.304	0.34	0.69	1.024 ± 0.264	1.020 ± 0.354	0.32	0.80	0.975 ± 0.329	1.021 ± 0.285	0.81	0.96
Total weight loss (1 y)	1.083 ± 0.270	1.075 ± 0.266	0.33	0.69	1.039 ± 0.341	1.046 ± 0.306	0.56	0.80	1.065 ± 0.302	1.032 ± 0.320	0.21	0.37
Total body fat (%) (1 y)	1.026 ± 0.303	0.989 ± 0.297	0.21	0.69	0.947 ± 0.331	1.035 ± 0.322	0.96	0.96	1.080 ± 0.360	1.002 ± 0.313	0.05	0.19
Δ Total body fat (%)	1.033 ± 0.308	1.025 ± 0.274	0.54	0.90	1.088 ± 0.286	1.039 ± 0.271	0.13	0.64	1.049 ± 0.285	1.016 ± 0.256	0.22	0.37
Fasting glucose (1 y)	1.033 ± 0.324	1.049 ± 0.340	0.72	0.98	1.099 ± 0.354	1.072 ± 0.325	0.26	0.80	1.104 ± 0.353	1.054 ± 0.330	0.08	0.19
Δ Fasting glucose (1 yr)	0.898 ± 0.473	0.987 ± 0.461	0.92	0.98	0.901 ± 0.488	0.951 ± 0.455	0.85	0.96	0.859 ± 0.462	0.954 ± 0.468	0.96	0.96
HbA1c (1 y)	1.098 ± 0.341	1.087 ± 0.356	0.21	0.69	1.026 ± 0.319	1.073 ± 0.350	0.90	0.96	1.086 ± 0.307	1.138 ± 0.316	0.87	0.96
Δ HbA1c (1 y)	1.111 ± 0.381	1.070 ± 0.344	0.21	0.69	1.111 ± 0.343	1.073 ± 0.348	0.48	0.80	1.092 ± 0.290	1.025 ± 0.363	0.07	0.19
MAF-5 (1 y)	0.975 ± 0.227	1.051 ± 0.219	0.98	0.98	1.031 ± 0.193	1.024 ± 0.179	0.49	0.80	1.007 ± 0.192	1.021 ± 0.211	0.70	0.96
Δ MAF-5 (1 y)	1.026 ± 0.183	1.077 ± 0.182	0.94	0.98	1.089 ± 0.185	1.024 ± 0.186	0.01	0.10	1.111 ± 0.183	1.043 ± 0.187	0.01	0.14

Conversely, the classification experiments, shown in Table 4, showed 17 significant input–target combinations. Notably, the highest performance was obtained for the prediction of the cross-surgical delta of fasting glucose using the postsurgical oral canon, yielding an AUC of 0.825 ± 0.225, which is in agreement with the results from Figure 5B, where three specific strains showed significant association with delta fasting glucose. Another notable result was the prediction of postsurgical TBF%, which could be predicted with an AUC of 0.808 ± 0.201. Finally, prediction of HbA1c using the postsurgical oral canon in the gut yielded an AUC of 0.775 ± 0.199. This concludes the set of experiments where the oral canon, measured in any of the available ways (presurgical, postsurgical, and log2 fold change), yielded a classification performance of an AUC over 0.75 on any of the dichotomized clinical characteristics.

**Table 4.** Classification results comparing each target value with a permuted version of itself. Results in bold font indicate statistically significant improvement from random.

	Presurgical				Postsurgical				Log2Fold change				Clinical			
Target	AUC	AUC (permuted)	*p*	*p*_adj	AUC	AUC (permuted)	*p*	*p*_adj	AUC	AUC (permuted)	*p*	*p*_adj	AUC	AUC (permuted)	*p*	*p*_adj
BMI (1 y)	0.540 ± 0.275	0.504 ± 0.296	0.19	0.32	0.592 ± 0.256	0.495 ± 0.291	4.6E-03	0.01	0.520 ± 0.261	0.478 ± 0.302	0.19	0.47	0.598 ± 0.245	0.510 ± 0.287	0.01	0.02
Total weight loss (1 y)	0.672 ± 0.229	0.455 ± 0.285	2.4E-08	2.4E-07	0.450 ± 0.257	0.502 ± 0.290	0.85	1.00	0.412 ± 0.253	0.488 ± 0.285	0.97	0.97	0.773 ± 0.224	0.490 ± 0.261	8.1E-13	4.0E-12
Total body fat (%) (1 y)	0.416 ± 0.264	0.478 ± 0.281	0.94	0.99	0.808 ± 0.201	0.497 ± 0.301	1.8E-13	1.8E-12	0.742 ± 0.189	0.477 ± 0.319	3.9E-10	3.9E-09	0.623 ± 0.212	0.522 ± 0.306	0.01	0.02
Δ Total body fat (%)	0.597 ± 0.272	0.494 ± 0.270	3.4E-03	0.02	0.423 ± 0.212	0.530 ± 0.265	1.00	1.00	0.675 ± 0.205	0.522 ± 0.272	1.5E-05	7.6E-05	0.524 ± 0.239	0.512 ± 0.295	0.40	0.50
Fasting glucose (1 y)	0.483 ± 0.240	0.520 ± 0.257	0.87	0.99	0.404 ± 0.282	0.460 ± 0.303	0.91	1.00	0.456 ± 0.250	0.466 ± 0.265	0.49	0.71	0.504 ± 0.264	0.512 ± 0.296	0.57	0.63
Δ Fasting glucose (1 y)	0.367 ± 0.306	0.475 ± 0.301	0.99	0.99	0.825 ± 0.225	0.400 ± 0.320	8.6E-05	4.3E-04	0.713 ± 0.288	0.562 ± 0.325	0.09	0.29	0.815 ± 0.247	0.552 ± 0.347	2.4E-08	6.1E-08
HbA1c (1 y)	0.458 ± 0.241	0.542 ± 0.278	0.84	0.99	0.775 ± 0.199	0.554 ± 0.259	4.2E-03	0.01	0.488 ± 0.264	0.613 ± 0.308	0.88	0.97	0.428 ± 0.290	0.467 ± 0.299	0.75	0.75
Δ HbA1c (1 y)	0.567 ± 0.195	0.425 ± 0.291	0.06	0.12	0.433 ± 0.271	0.487 ± 0.287	0.73	1.00	0.454 ± 0.293	0.546 ± 0.347	0.83	0.97	0.748 ± 0.231	0.524 ± 0.276	1.1E-08	3.8E-08
MAF-5 (1 y)	0.721 ± 0.248	0.475 ± 0.249	0.01	0.02	0.558 ± 0.192	0.475 ± 0.285	0.14	0.28	0.487 ± 0.218	0.454 ± 0.244	0.32	0.65	0.805 ± 0.203	0.510 ± 0.235	2.4E-16	2.4E-15
Δ MAF-5 (1 y)	0.608 ± 0.261	0.429 ± 0.280	0.01	0.04	0.483 ± 0.223	0.508 ± 0.340	0.63	1.00	0.442 ± 0.243	0.442 ± 0.260	0.44	0.71	0.713 ± 0.233	0.520 ± 0.273	8.7E-07	1.7E-06

The classification experiments for the postsurgical canon were repeated with the presurgical T2D status and PPI use added as known confounders. This addition increased the predictive performance of the glucose delta to an AUC of 0.937 ± 0.134. The HbA1c and total TBF% experiments yielded lower performance, namely 0.758 ± 0.134 and 0.733 ± 0.243 for TBF% and HbA1c, respectively.

## Discussion

This study set out to test to what extent RYGB reshapes the oral and gut microbiomes in a way that promotes expansion of oral strains in the distal gut, and whether such strain-level dynamics relate to metabolic outcomes. To that end, we defined a cohort-specific, strain-level canonical oral microbiota and tracked its presence, abundance, and compositional structure in paired oral and fecal samples before and 12 months after surgery, alongside exploratory links with clinical phenotypes and predictive modeling of postsurgical metabolic markers. Although an increase in oral strains in the gut after RYGB is among the most consistently reported changes in the microbiota after surgery, direct evidence of oral-to-gut microbial co-occurrence at the strain level has been lacking.^4^ ^,^ ^9^ ^,^ ^15-18^ By leveraging strain-level profiling,^31^ our work provides novel evidence of oral-to-gut microbial transfer in the context of RYGB.

Several consistent patterns emerged. In the oral cavity, strain richness increased after surgery while overall diversity remained stable, with a modest but coherent community shift and preservation of individual signatures. In the gut, strains from the canonical oral microbiota became more numerous and collectively more abundant after RYGB, with a marked convergence of community composition across individuals. In this regard, we found a clear and strong postoperative community, where the dissimilarity between the postoperative community was smaller than the intraspecific cross-surgical dissimilarity between samples. Within the set of orally present taxa in the gut, 24 out of 60 shared strains significantly increased, and these expanding strains represented most of the oral-canon abundance postsurgery. Cross-site correlations for multiple strains support coordinated changes between oral and gut niches.

In this work, we adjusted the abundance of the oral strains in the gut by PPI use and T2D status when evaluating the association with clinical characteristics. Both PPI and T2D are shown to be associated with substantial microbial shifts.^36^ ^,^ ^37^ Nevertheless, this work focusses mainly on associations which were only present both with, and without adjustment for confounders. In this manner, the robustness of the found associations is maximized given the available data in this study.

Our findings demonstrate that the expansion of oral-origin strains in the gut after RYGB is not only a reproducible ecological phenomenon but may also indicate clinical relevance. The significant associations between the abundance and fold change of the oral canon strains and key metabolic parameters, such as FIB-4, ASAT, and fasting glucose, suggest that the postsurgical enrichment of these strains in the gut may contribute to hepatic and glycemic outcomes. Notably, the link between higher postsurgical abundance and poorer hepatic metabolic health highlights the need to further investigate the functional roles of these strains in host metabolism.

In the context of obesity and glycemic traits, our data suggest that a more abundant presurgical oral canon in the gut identifies individuals who undergo particularly strong metabolic remodeling after RYGB. Higher presurgical abundances of several canonical oral strains, including *Actinomyces oris*, *Actinomyces graevenitzii*, *Gemella sanguinis*, *Rothia mucilaginosa*, and *Streptococcus australis*, were associated with larger reductions in fasting glucose, consistent with an obesity-associated, oral-like community that is especially responsive to the profound weight loss and hormonal changes after surgery. This interpretation is supported by independent stool and saliva studies that report enriched abundance of *Actinomyces oris* and *Rothia mucilaginosa* in the gut microbiota of people with obesity compared with lean control individuals,^38^ ^,^ ^39^ and depletion of *Streptococcus australis* in the gut microbiota of people with obesity.^38^ Together, these findings indicate that these taxa mark an obesity-linked microbiome configuration that can be substantially reshaped when energy balance is surgically disrupted.

With respect to glycemic remodeling more broadly, we observed that both baseline and postsurgical abundances of several oral canon members related to changes in fasting glucose and HbA1c. Presurgical levels of *Actinomyces oris*, *Actinomyces graevenitzii*, *Gemella sanguinis*, *Rothia mucilaginosa*, and *Streptococcus australis* in the gut were negatively associated with changes in fasting glucose, indicating that higher baseline carriage predicted greater reductions in glycaemia after RYGB. In the postsurgical setting, fecal *Actinobaculum sp. oral taxon 183* and *Veillonella parvula* showed negative associations with delta fasting glucose, whereas fecal *Isoptericola variabilis* was positively associated with presurgical HbA1c and negatively with delta HbA1c, consistent with larger HbA1c improvements in those with worse baseline control. These findings are in line with reports that fecal *Rothia mucilaginosa* is enriched in the stool of individuals with type 2 diabetes compared with healthy controls^39^ and that *Veillonella* species, including *Veillonella atypica* and *Veillonella parvula*, correlate positively with glucagon-like peptide 1 (GLP-1) responses and negatively with body weight and waist circumference in RYGB.^40^ Within this framework, the very high predictive power of the postsurgical oral canon on change in fasting blood glucose, yielding an average AUC of 0.937, further underscores the link between the influx of orally derived taxa and improvements in glucose metabolism.

Several of the same oral strains in the gut were also linked to markers of liver injury and fibrosis, which points to a shared oral‒hepatic axis that is relevant for MASLD. Postsurgical log2 fold changes in fecal *Actinomyces oris*, *Streptococcus parasanguinis*, and *Streptococcus salivarius* were positively associated with ASAT, and *Veillonella parvula* was positively associated with changes in FIB-4, indicating that expansion of these strains after surgery coincided with less favorable hepatic trajectories. This pattern is consistent with cirrhosis studies in which fecal Streptococcus salivarius, *Streptococcus parasanguinis*, and V*eillonella* species, including *Veillonella parvula*, are enriched in stool from patients with liver cirrhosis compared with healthy controls,^41^ and with interventional work in MASLD where combined metabolic activators reduced *Actinobaculum sp. oral taxon 183*, *Rothia mucilaginosa*, and *Streptococcus parasanguinis* in both the oral cavity and stool in parallel with improvements in liver-related metabolic parameters.^42^ Although these associations do not establish causality, they support the idea that fecal abundance of specific oral-derived taxa participates in MASLD-related hepatic perturbations and that modulation of their abundance may accompany therapeutic improvement.

Mechanistically, these findings align with an RYGB-induced lack of ecological filter that favors oral, facultative anaerobes in the lower gut, consistent with changes in luminal oxygen tension, pH, transit time, and bile acid pools.^12^ ^,^ ^43^ ^,^ ^44^ The strong postsurgical convergence of the oral canon in the gut suggests a reproducible post-RYGB niche. The inverse relationship between presurgical abundance and postsurgical fold change further points to saturation or ceiling effects, in which higher transfer of orally derived strains presurgery limits further expansion postsurgery.

This work has limitations. First, the sample size is modest and from a single-center study, and sampling was limited to two time points, precluding a fine-grained temporal view of colonization dynamics. While strain-level profiling reduces taxonomic ambiguity, it remains susceptible to limits in reference coverage and depth; assembly-based corroboration would add robustness. Secondly, only tongue-swab samples were used to represent the oral microbiota, whereas the oral cavity comprises multiple distinct niches with different community compositions.^45^ Third, we did not collect detailed postoperative dietary intake data and were therefore unable to adjust for differences in diet after RYGB. Although all participants received standardized dietary counseling as part of routine bariatric care,^46^ interindividual variation in adherence and habitual food choices may have contributed to the observed variability in oral and fecal microbiota and metabolite profiles. Fourth, only six participants had type 2 diabetes at baseline, so our study is underpowered for subgroup analyzes of diabetes status; references to glycemic remodeling should therefore be interpreted primarily in terms of continuous glycemic markers (fasting glucose and HbA1c) in the overall cohort rather than disease-specific effects. Finally, all associations with clinical outcomes were exploratory and require replication with appropriate multiplicity control.

Future studies should prioritize (I) dense longitudinal sampling to resolve early postoperative dynamics; (II) multi-site sampling of the oral microbiota to obtain a more complete view of the strains occurring in the oral cavity; (III) *in vitro* assays (e.g., strain isolation, coculture, epithelial and immune models) to test mechanisms and fitness of oral-derived strains under post-RYGB conditions; and (IV) mouse experiments in an RYGB model combined with targeted probiotic administration of candidate oral taxa to establish causality and therapeutic modifiability.

In conclusion, RYGB is associated with a reproducible, strain-level enrichment of oral, facultative anaerobic taxa in the gut and a convergence of the oral-canon community postsurgery. These changes show preliminary links to glycemic traits, including fasting glucose and HbA1c, and carry prognostic signal for postsurgical fasting glucose. Together, the results support a contributory role of oral-derived strains in the metabolic remodeling after RYGB and motivate mechanistic, longitudinal, and interventional work toward microbiome-informed care.

## Supplementary Material

supplemental_tables.docx Supplemental Material

## Data Availability

Fecal metagenomic shotgun data have been deposited in the European Nucleotide Archive (ENA; https://www.ebi.ac.uk/ena/browser/view/ERP132222).

## Abbreviations

ASAT: Aspartate aminotransferase
AUC: Area under the curve
BA: Bile acids
BH: Benjamini–Hochberg
BMI: Body mass index
CI: Confidence interval
DNA: Deoxyribonucleic acid
FIB-4: Fibrosis-4 index
HbA1c: Glycated hemoglobin
IHMS: International Human Microbiome Standards
MAE: Mean absolute error
MAFLD/MASLD: Metabolic dysfunction-associated steatotic liver disease
ML: Machine learning
NIT: Noninvasive test
PCoA: Principal coordinates analysis
PPI: Proton pump inhibitor
R2: Coefficient of determination
ROC: Receiver operating characteristic
RYGB: Roux-en-Y gastric bypass
SCFA: Short-chain fatty acid
SD: Standard deviation
SGB: Species-level genome bin
T2D: Type 2 diabetes
TBF%: Total body fat percentage
TWL%: Total weight loss percentage
