# Supplementary material for: Oral–gut strain sharing after Roux-en-Y gastric bypass: A canonical oral microbiome signature in the gut linked to hepatic and glycemic remodeling
Source: Gut Microbes Rep. 2026 Jul 22;3(1):2693432. doi: 10.1080/29933935.2026.2693432 (PMC13393230; doi:10.1080/29933935.2026.2693432)
Supplement: supplemental_tables.docx — Supplemental Material [file KGMR_A_2693432_SM1307.docx]

*Supplemental table 1: use of blood pressure lowering & glucose lowering medication by timepoint*

| **Medication type** | **Baseline  n (%)** | **1-year follow-up  n (%)** |
| --- | --- | --- |
| blood pressure lowering | 10 (40%) | 7 (28%) |
| glucose lowering | 5 (20%) | 0 (0%) |

*Supplemental table 2: Significant associations between the presurgical abundance of the strains in the oral canon in the gut and clinical characteristics after surgery and cross-surgical delta*

|  | | | **Δ fasting glucose (1yr)** | | | **ASAT (1yr)** | | | **MAF-5 (1yr)** | | |
| --- | --- | --- | --- | --- | --- | --- | --- | --- | --- | --- | --- |
|  | **beta** | **p** | | **p_adj_** | **beta** | **p** | **p_adj_** | **beta** | | **p** | **p_adj_** |
| *Streptococcus parasanguinis* SGB8071 | -1.051 | 0.073 | | 0.136 | -2.582 | 0.432 | 0.662 | -0.156 | | 0.804 | 1.000 |
| *Streptococcus salivarius* SGB800 | 0.180 | 0.632 | | 0.632 | -0.939 | 0.682 | 0.792 | -0.041 | | 0.925 | 1.000 |
| *Actinomyces sp ICM47 S*GB1716 | -9.269 | 0.202 | | 0.253 | -168.347 | **0.002** | **0.015** | -31.917 | | **0.002** | **0.031** |
| *Actinomyces oris* SGB1587 | -31.073 | **0.005** | | **0.027** | -303.074 | **0.002** | **0.015** | -45.108 | | **0.020** | 0.102 |
| *Streptococcus australis* SGB8059 | -7.567 | **0.014** | | **0.042** | -17.527 | 0.377 | 0.662 | -1.852 | | 0.627 | 0.940 |
| *Actinomyces graevenitzii* SGB17130 | -186.877 | **0.002** | | **0.024** | -602.590 | 0.286 | 0.662 | -100.254 | | 0.353 | 0.662 |
| *Streptococcus mitis* SGB8168 | -39.882 | 0.129 | | 0.204 | 2.753 | 0.978 | 0.978 | 6.820 | | 0.715 | 0.975 |
| *Rothia mucilaginosa* SGB1697 | -36.297 | **0.010** | | **0.039** | -27.216 | 0.686 | 0.792 | 0.105 | | 0.993 | 1.000 |
| *Granulicatella sp* UMB5615A SGB8255 | -45.633 | 0.200 | | 0.253 | 13.669 | 0.940 | 0.978 | 0.015 | | 1.000 | 1.000 |
| *Gemella sanguinis* SGB729 | -73.633 | **0.003** | | **0.024** | -596.812 | **0.005** | **0.024** | -75.843 | | 0.079 | 0.297 |
| *Schaalia odontolytica* SGB1716 | -8.622 | 0.065 | | 0.136 | -89.118 | **0.024** | 0.090 | -17.851 | | **0.017** | 0.102 |
| *Schaalia* SGB1715 | 4.709 | 0.595 | | 0.632 | 4.231 | 0.442 | 0.662 | 1.007 | | 0.333 | 0.662 |
| *Lancefieldella parvula* SGB96 | -88.605 | 0.136 | | 0.204 | -735.526 | 0.171 | 0.513 | -109.872 | | 0.288 | 0.662 |
|  | | | | | | *cells shown in* ***Bold*** *indicate a signigicant association (p-value < 0.05)* | | | | | |

*Supplemental table 3: Significant associations between the postsurgical oral canon in the gut and clinical characteristics after surgery and cross-surgical delta.*

|  | **Fasting Glucose** | | | **Fasting Glucose (1yr)** | | | **Δ fasting glucose (1yr)** | | | **HbA1c** | | | **Δ HbA1c (1yr)** | | | **FIB-4 (1yr)** | | | **Δ FIB-4 (1yr)** | | |
| --- | --- | --- | --- | --- | --- | --- | --- | --- | --- | --- | --- | --- | --- | --- | --- | --- | --- | --- | --- | --- | --- |
|  | **beta** | **p** | **p_adj_** | **beta** | **p** | **p_adj_** | **beta** | **p** | **p_adj_** | **beta** | **p** | **p_adj_** | **beta** | **p** | **p_adj_** | **beta** | **p** | **p_adj_** | **beta** | **p** | **p_adj_** |
| *Streptococcus salivarius* SGB800 | -0.006 | 0.934 | 0.961 | -0.015 | 0.561 | 0.945 | 0.127 | **0.004** | **0.048** | -0.024 | 0.619 | 0.926 | -0.126 | 0.333 | 0.983 | 0.113 | **0.031** | 0.111 | 0.023 | 0.348 | 0.545 |
| *Actinobaculum sp oral taxon 183* SGB1589 | -21.343 | 0.622 | 0.899 | -9.713 | 0.515 | 0.930 | -82.539 | **0.002** | **0.034** | -20.156 | 0.468 | 0.926 | 21.290 | 0.473 | 0.983 | -26.118 | **0.027** | 0.107 | -7.045 | 0.199 | 0.341 |
| *Veillonella parvula* SGB693 | 26.684 | **0.001** | **0.010** | -2.167 | 0.477 | 0.930 | 12.160 | 0.092 | 0.368 | 14.626 | **0.005** | 0.090 | -17.140 | **0.002** | **0.032** | 6.104 | **0.012** | 0.084 | 3.467 | **0.001** | **0.019** |
| *Streptococcus vestibularis* SGB800 | -1.714 | 0.392 | 0.899 | -0.663 | 0.338 | 0.868 | 4.710 | **0.000** | **0.000** | 0.050 | 0.969 | 0.998 | -0.311 | 0.842 | 0.983 | 0.778 | 0.229 | 0.413 | -0.178 | 0.542 | 0.749 |
| *Streptococcus gordonii* SGB8053 | 2.565 | 0.389 | 0.899 | 0.023 | 0.982 | 0.982 | 1.322 | 0.501 | 0.693 | 2.327 | 0.222 | 0.926 | -2.178 | 0.333 | 0.983 | 2.677 | **0.001** | **0.047** | 0.854 | **0.033** | 0.162 |
| *Isoptericola variabilis* SGB1715 | 12.902 | **0.000** | **0.001** | -0.366 | 0.780 | 0.980 | 5.014 | 0.329 | 0.648 | 9.325 | **0.000** | **0.000** | -8.413 | **0.000** | **0.002** | 1.632 | 0.131 | 0.294 | 0.867 | 0.066 | 0.215 |
| *Actinomyces sp oral taxon* 448 SGB1587 | -19.622 | 0.643 | 0.899 | -44.774 | **0.000** | **0.016** | 5.766 | 0.841 | 0.918 | -13.734 | 0.615 | 0.926 | 16.896 | 0.623 | 0.983 | -14.243 | 0.323 | 0.481 | -10.205 | 0.101 | 0.242 |
| *Veillonella atypica* SGB693 | -0.079 | 0.840 | 0.941 | -0.075 | 0.577 | 0.945 | 0.564 | **0.016** | 0.112 | -0.254 | 0.308 | 0.926 | -4.347 | 0.286 | 0.983 | 3.944 | **0.014** | 0.084 | 1.936 | **0.005** | 0.097 |
|  | *cells shown in* ***Bold*** *indicate a signigicant association (p-value < 0.05)* | | | | | | | | | | | | | | | | | | | | |

*Supplemental table 4: Significant associations between the log2fold change of the oral canon in the gut and clinical characteristics after surgery and cross-surgical delta.*

|  | **ASAT (1yr)** | | | **FIB-4 (1yr)** | | | | **Δ FIB-4 (1yr)** | | | |  |  |
| --- | --- | --- | --- | --- | --- | --- | --- | --- | --- | --- | --- | --- | --- |
|  | **beta** | **p** | **p_adj_** | | **beta** | **p** | **p_adj_** | | **beta** | **p** | **p_adj_** | |  |
| *Streptococcus parasanguinis* SGB8071 | 0.770 | **0.003** | **0.016** | | 0.017 | 0.282 | 0.380 | | 0.008 | 0.235 | 0.352 | |  |
| *Streptococcus salivarius* SGB800 | 0.677 | **0.003** | **0.016** | | 0.014 | 0.304 | 0.380 | | 0.007 | 0.244 | 0.352 | |  |
| *Actinomyces oris* SGB1587 | 1.006 | **0.002** | **0.016** | | 0.031 | 0.116 | 0.289 | | 0.019 | **0.026** | 0.128 | |  |
| *Streptococcus australis* SGB8059 | 0.551 | **0.009** | **0.035** | | 0.015 | 0.223 | 0.380 | | 0.005 | 0.408 | 0.470 | |  |
| *Actinomyces graevenitzii* SGB17130 | 0.386 | 0.308 | 0.340 | | 0.055 | **0.002** | **0.032** | | 0.017 | 0.050 | 0.180 | |  |
| *Granulicatella sp UMB5615A* SGB8255 | 0.661 | 0.052 | 0.098 | | 0.037 | **0.042** | 0.158 | | 0.022 | **0.004** | 0.062 | |  |
|  | *cells shown in* ***Bold*** *indicate a signigicant association (p-value < 0.05)* | | | | | | | | | | | | |
